# Taxonomic and functional profiling of the vulvar microbiome indicates variations related to ecological signatures, aging, and health status

**DOI:** 10.3389/fmicb.2025.1633147

**Published:** 2025-09-19

**Authors:** Arnout Mieremet, Michelle van der Wurff, Lisa Pagan, Edgar Ferrer-González, Jin Seo, Frank H. J. Schuren

**Affiliations:** ^1^Department of Microbiology and Systems Biology, Netherlands Organization for Applied Scientific Research (TNO), Leiden, Netherlands; ^2^Centre for Human Drug Research (CHDR), Leiden, Netherlands; ^3^Reckitt Benckiser LLC, Montvale, NJ, United States

**Keywords:** vulvar microbiome, metagenomic sequencing, taxonomy, functional profiling, vulvar lichen sclerosus, vulvar diseases

## Abstract

**Introduction:**

The vulvar microbiome is adjacent to that of the skin and the vagina and connects microbiomes present on a stratified epithelial barrier to that of a mucosal barrier. Yet, the characterization of the microbiome in the vulvar region of the body is understudied, although dysbiosis in the microbiome of the skin or vagina have been linked to impairments in women’s health.

**Methods:**

To better understand the role of the vulvar microbiome during healthy aging or during presentation of vulvar diseases, we analyzed the vulvar microbiome by shotgun metagenomic sequencing on composition at species level and for functional capacity. This was performed in a large population enrolled in the Vulvar Microbiome Leiden Cohort (VMLC), including a total of 58 healthy women in a broad age range (22–82 years). Moreover, we analyzed vulvar microbiome derived from 9 participants presenting a vulvar disease, including vulvar lichen sclerosus (LS; *N* = 6), or high-grade squamous intraepithelial lesion (HSIL; *N* = 3).

**Results:**

Compositional analyses showed a skin-, vagina-, or multispecies mixture- dominant bacterial signature, which revealed differences in the alpha diversity and functional capacity of the microbiome. Upon aging the presence of *Lactobacillus iners*, *L. crispatus*, and *L. gasseri* in the vulvar microbiome shifted toward reduction. In the microbiome of individuals with a vulvar disease, higher abundance of *Staphylococcus hominis*, *Micrococcus luteus*, *Corynebacterium amycolatum*, and *Corynebacterium simulans* was detected, and an altered functional capacity for the L-histidine pathway.

**Discussion:**

In conclusion, we identified variations in microbial taxa and functional capacities in the vulvar microbiome that are associated with age and disease (LS and HSIL), which can be targeted to develop microbiome-based vulvar therapies promoting women’s health.

## 1 Introduction

The microbiome is a highly complex ecosystem consisting of bacteria, viruses, archaea, and eukaryotes that cover epithelial tissues in the human body. Continuous and versatile interactions take place within the microbiome or bidirectionally between the microbiome and the host. These interactions shape the composition and functionality of the microbiome. Moreover, various aspects in the physiology of the human host can influence the microbiota. Niche-specific microbiomes can be identified based on the nature of the epithelial tissue, for example the skin (Byrd et al., 2018) and vagina (Ma et al., 2012). Yet, additional factors play a role in orchestrating the microbial composition, which can be related to biophysical characteristics, such as age (Alkema et al., 2021; Verstraelen et al., 2022), and/or health status (White et al., 2011; Chng et al., 2016). The compositions of the microbiome have been characterized for major organs or body sites such as the gut (Shanahan et al., 2021), skin (Byrd et al., 2018; Saheb Kashaf et al., 2022), and vagina (Ma et al., 2012; Lebeer et al., 2023; Norenhag et al., 2024). Yet, the microbiome composition of the vulva, which form the outer part of the female genitals and connects the stratified epithelium of the skin with the mucosal epithelium of the vagina, is an untapped area that has not been characterized in high taxonomic resolution.

The vulvar microenvironment is a highly complex system inhabited by niche-specific communities of bacteria and other micro-organisms (Larsen and Monif, 2001). A balanced vulvar microbiome plays a vital role in the prevention of diseases as a result of extensive host-microbe and microbe-microbe interactions (Nardini et al., 2016). Moreover, continuous host-microbe interactions lead to trained responses of human vulvar epithelial and immune cells upon external or internal stimuli. Homeostatic processes are affected by alterations in the composition of the microbiome. In fact, vulvar microbiome dysbiosis is associated with a higher risk to develop diseases in the female genital tract (Pybus and Onderdonk, 1999). Yet, due to the complexity of the host-microbe and microbe-microbe interactions occurring in the vulvar ecosystem, it is currently unknown what defines a healthy (Joos et al., 2024) normal vulvar microbiome and what drives vulvar microbiome dysbiosis.

The role of the vulvar microbiome in health and disease is scarcely studied. Only a few studies have been published until now, which often lack analytical sensitivity to determine microbiota composition at species level (Pagan et al., 2021, 2023; Ma et al., 2024; Pyle et al., 2024). These studies suggested that the vulvar microbiome contains bacteria associated with skin, vagina (Amabebe and Anumba, 2018), and possibly with the intestine. However, the findings were based on a small number of participants per study. Furthermore, the potential effects of intrinsic aging and health status has not been examined in high taxonomic resolution.

Our aim was to investigate the vulvar microbiome at species level using shotgun metagenomic sequencing for compositional and functional profiles. Using shotgun metagenomics, species-level taxonomic resolution and functional insights can be obtained, which are main advantages as compared to traditional 16S rRNA gene sequencing approaches (Ranjan et al., 2016; Wensel et al., 2022). We characterized the vulvar microbiome of 58 healthy women in a broad age range (22–82 years) and we analyzed the vulvar microbiome of 6 women with vulvar lichen sclerosus (LS), and 3 women with vulvar high-grade squamous intraepithelial lesion (HSIL). We demonstrated that the vulvar microbiome can be classified based on its composition as skin-, vagina-, or multispecies mixture- dominant bacterial signature.

## 2 Materials and methods

### 2.1 Cohort description

The samples collected in the vulvar microbiome aging cohort (TNO; *n* = 49) are described in this manuscript. The vulvar microbiome cohort (CHDR; *n* = 18) has been described by Pagan et al. (2023). A merged dataset of both cohorts is defined as the Vulvar Microbiome Leiden Cohort (VMLC), which covers a total of 67 women of which 58 were healthy, 3 with vulvar HSIL, and 6 with LS.

### 2.2 Sample collection vulvar swabs

Vulvar swabs were collected by one trained gynecologist to minimize sampling variation during collection. Samples were anonymously collected non-invasively at one time point during routine gynecological visits following verbal consent. According to the ethical standards described in the Medical Research Involving Human Subjects Act (in Dutch: Wet Medisch-Wetenschappelijk onderzoek met mensen, WMO) this study is classified as non-WMO research in The Netherlands (CCMO, 2019; Scott et al., 2020). The protocol used for this was as follows: Zymo DNA-RNA shield Collection Tube w-Swabs were pre-wetted with the swab solution. Swabs were obtained by swiping vigorously on the chosen vulvar location for 30 s (approximate skin surface area 4 cm × 4 cm or limited to the lesion), after which the swabs were transferred to the collection tubes and stored at −80 °C.

### 2.3 DNA isolation

Each well of a 96-deep-well plate containing 50 μL microbiome sample was supplemented with 500 μL washed zirconium beads (0.1 mm; BioSpec products, Bartlesville, USA) in Milli-Q water and 800 μL CD1 solution from the DNeasy 96 Powersoil Pro QIAcube HT Kit (Qiagen). The plate was sealed and homogenized 4 min. Subsequently, the plates were centrifuged at 3000 × *g* for 6 min. Then, 600 μL of supernatant was transferred into a fresh plate containing 300 μL CD2 solution from the DNeasy 96 Powersoil Pro QIAcube HT Kit (Qiagen) and mixed by pipetting up and down three times. After centrifugation of the plate at 3000 × *g* for 6 min, 550 μL of the supernatant was transferred to an S-block (Qiagen). From this point on, the QIAcube Connect instrument (Qiagen, Germantown, MD) was used and extraction protocols were followed according to manufacturer’s instructions. Purified DNA was stored at −20 °C in EB buffer (Qiagen).

### 2.4 Vulvar swab sequencing analyses

Shotgun metagenomic sequencing of samples in the TNO cohort was performed according to protocols provided by Illumina (Illumina, San Diego, USA) also previously described (Wiese et al., 2024) and for CHDR cohort described by Pagan et al. (2023). Sequencing outcomes of both cohort were processed in the same analysis pipeline. Shotgun metagenomic sequence analysis and taxonomic and functional classification was performed by the bioBakery 3 platform (Beghini et al., 2021) including quality control with host DNA filter step (KneadData version v0.10.0), taxonomic profiling (MetaPhlAn version 3.0), and functional profiling (HUMAnN version v3.0.1). The bacterial ChocoPhlAn database version mpa_v31_CHOCOPhlAn_2010901 and metabolic pathway MetaCyc database version v24 was used for species and enzyme classification. To determine niche-specific dominant signatures, skin and vagina specific species were selected that are (nearly) exclusively present in one of both ecological niches. For skin, *Cutibacterium* and *Staphylococcus* species were selected (Grice and Segre, 2011; Townsend and Kalan, 2023), while *Gardnerella*, *Lactobacillus, Prevotella*, *Atopobium*, *Finegoldia* and *Ureaplasma* species were selected for the vagina (Moosa et al., 2020; Dubé-Zinatelli et al., 2024). We do not exclude that bacteria from those genera never occur in the other niches, but these are generally at much higher levels in their own niche.

### 2.5 Statistical analysis

Taxonomic and functional count tables derived from shotgun metagenomic data were obtained using MetaPhlAn and HUMAnN and subsequently imported as phyloseq objects (McMurdie and Holmes, 2013). Taxonomic data was pre-processed to retain only bacterial species and exclude low count species by cumulative counts (threshold: 0.05%) beta diversity analysis. For functional analysis, enzymes present in at least 10% of the samples were selected, while redundant enzymes were removed. Additionally, two samples (HGHNYDSX3_104965-001-104_GATGCACT-ACACCAGT and HGHNYDSX3_104965-001-092_TAAGTGGC-ATCCGTTG) were excluded due to low read counts (≤20.000), and one sample (NS-35-sample-15_S15_L001) was excluded due to missing metadata. Alpha diversity was assessed using the Shannon index tested for significance by Dunn’s test. PCA was tested using one-way PERMANOVA with correction for multiple testing, performed with the multilevel pairwise comparison using adonis2 of the R package “vegan.” Beta diversity was characterized through principal component analysis (PCA) with centered log-ratio (CLR) transformation with the vegan package (Oksanen et al., 2019). Differential abundance analysis of species was conducted using DESeq2 (Love et al., 2014), incorporating a pseudocount of 1, with comparisons based on age or disease state relative to a Q1_Q2 (age) or healthy reference. Enzymatic differentially abundant features were identified using Limma (Ritchie et al., 2015). Effect sizes were visualized through heatmaps with log2 fold changes, with statistical significance determined at an adjusted *p* < 0.05. Relative abundance bar graphs were used to highlight the most prevalent taxonomic units. Taxonomic data was pre-processed to retain only bacterial species and exclude low count species by cumulative counts (threshold: 0.05%) beta diversity analysis. For visualization purposes, PCA loadings were extracted and scaled (lengthened) to enhance interpretability in the biplot. All visualizations were generated using ggplot (Wickham and Sievert, 2009) in R.

## 3 Results

### 3.1 Taxonomic profiling on species level of the healthy vulvar microbiome

To determine the composition of the vulvar microbiome in healthy women, vulvar swabs were collected, extracted, and analyzed with shotgun metagenomic sequencing. This was performed in the Vulvar Microbiome Leiden Cohort (VMLC), which is a combination of samples collected in this study (referred as TNO) and samples collected in the study of Pagan et al. (2023) (referred as CHDR). Examination of the vulvar microbiomes in these cohorts was performed by shotgun metagenomic sequencing that facilitated analysis of entire genes of all organisms. Genetic material of the vulvar microbiome was obtained by collecting microbes via a swabbing procedure. During sample collection, genetic material of the human host was also obtained that potentially interferes with the sequencing analysis. Therefore, an evaluation of the level of host DNA in the sample was performed. This showed the presence of host DNA with clear differences between samples. The number of reads on host DNA did not lead to interference of the vulvar microbiome analyses (Supplementary Table 1).

To merge both cohorts into a combined dataset, all sequencing data was analyzed in the same pipeline, followed by an unsupervised cohort comparative analysis (Supplementary Figure 1A). This showed no substantial differences between cohorts and subgroups. Furthermore, a comparative analysis for the Shannon diversity index was performed on samples derived from healthy women, which revealed comparable average and variance of samples in either the CHDR or the TNO cohort (Supplementary Figure 1B). Therefore we continued with merging of data into the VMLC, which includes a total of 58 healthy women with a broad age range (mean age in years 48.9, range 22–82).

**FIGURE 1:**
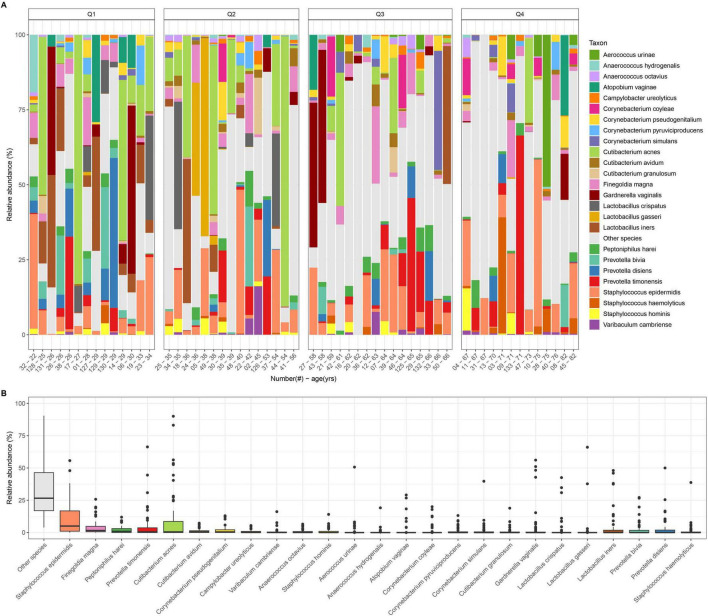
Taxonomic profiling of the vulvar microbiome in healthy women. **(A)** The relative abundance of bacterial species in the vulvar microbiome. Samples are plotted by age of the subjects on the *x*-axis and clustered in quartiles based on the age [Q1 (22–34), Q2 (35–56), Q3 (58–66), and Q4 (67–82)], in healthy cohort of *n* = 58 subjects. When subjects had identical age, these were included in the younger age quantile. **(B)** Box plot of top 25 bacterial species in the healthy vulvar microbiome, and species beyond top 25 are indicated cumulatively in gray as other species. Microbes that are in the top 25 overall in presence are indicated by colored bars.

The composition of the healthy vulvar microbiome was investigated by the relative abundance in the vulvar microbiome (Figure 1A). The bacteria that were most abundant at species level varied across samples. The overall highest median relative abundances were detected for *Staphylococcus epidermidis*, *Finegoldia magna*, *Peptoniphilus harei*, *Prevotella timonensis*, and *Cutibaterium acnes* (Figure 1B).

### 3.2 Vulvar microbiome can present a skin or vaginal dominant signature in healthy women

**FIGURE 2:**
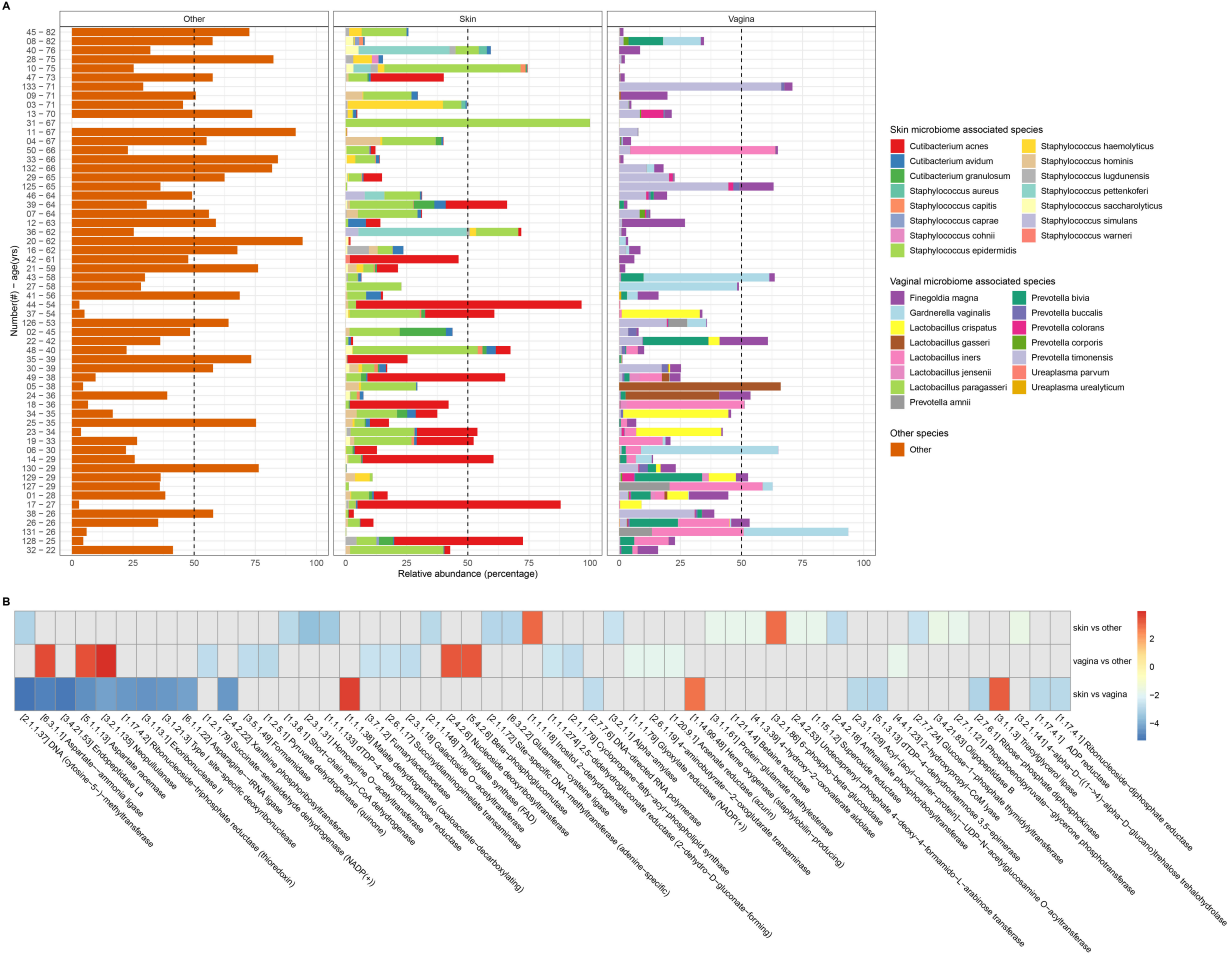
Classification of the vulvar microbiome based on ecological signatures. **(A)** Bar plot represents the vulvar microbiome composition based on ecological signatures. A skin- or vagina- signature was defined as ≥50% of the microbes in the vulvar microbiome are identified as skin- or vagina- associated. The threshold per sample is indicated by a dashed line. Species classified as skin- or vagina- associated are presented in the legend. Microbiomes without a skin or vaginal signature are indicated as other. **(B)** Comparative analysis of the functional capacities in the vulvar microbiome for the three vulvar microbiome types. Enzyme names and Enzyme Commission (EC) identifier codes are indicated in *X*-axis. Heat map indicator is shown. Cohort size *n* = 58.

The vulvar microbiome forms a unique interface by bridging the skin microbiome with the vaginal mucosal microbiome. Therefore, we further investigated the influence of both microbiomes on that found in the vulvar ecosystem. To do so, we indicated bacteria which are mainly found in the skin (Grice and Segre, 2011; Townsend and Kalan, 2023) or in the vagina (Moosa et al., 2020; Dubé-Zinatelli et al., 2024) to establish a microbial signature of the vulvar microbiome for each sample. Both typical skin as well as vaginal microbes have been observed in the vulvar microbiome, with clear variations between individual samples (Figure 2A). Skin- or vagina- signatures were defined when ≥50% of the microbes in the vulvar microbiome were classified as typical skin or vaginal. In 24% (14 out of 58) of the samples, a skin- dominant microbiome signature was identified, whereas in 22% (13 out of 58) of the samples a vagina- dominant microbiome signature was observed. In skin- dominant vulvar microbiomes, the presence of *C. acnes* or *S. epidermidis* was highest. In vagina- dominant vulvar microbiomes, the presence of *G. vaginalis*, *F. magna*, or *L. iners* was highest. The other samples were more indicative of a multispecies mixture of species from both the vaginal and skin microbiome, forming a vulvar signature in 53% (31 out of 58) of the individuals (Supplementary Table 2). The alpha diversity of the multispecies mixture vulvar microbiome was significantly higher as compared to skin- or vagina-dominant signatures (Supplementary Figure 2A). A beta-diversity based PCA analysis showed significant differences between all signatures, except between vagina- versus other subgroups (Supplementary Figure 2B). Species indicative for the skin- or vagina- dominant vulvar microbiome directs the PCA plots to a opposite directions, whereas the multispecies mixture showed no specific directionality (Supplementary Figures 2C–E). These findings suggest that the ecological origin in the vulvar ecosystem can be diverse, yet they exhibit a signature that is skin-, vagina-, or multispecies mixture- dominant.

Subsequently, we investigated whether the signatures affect the functional capacity of the microbiome composition, we analyzed the genetic material for encoding functional enzymes, revealing substantial differences between vulvar and vaginal dominant signatures (Figure 2B). This revealed that there is a substantial difference specific to the vulvar microbiome with a vagina- dominant signature regarding the aspartate pathway (Aspartate–ammonia ligase [EC6.3.1.1] and Aspartate racemase [EC5.1.1.13]) compared to other signatures.

### 3.3 The composition of the vulvar microbiome in relation to aging

**FIGURE 3:**
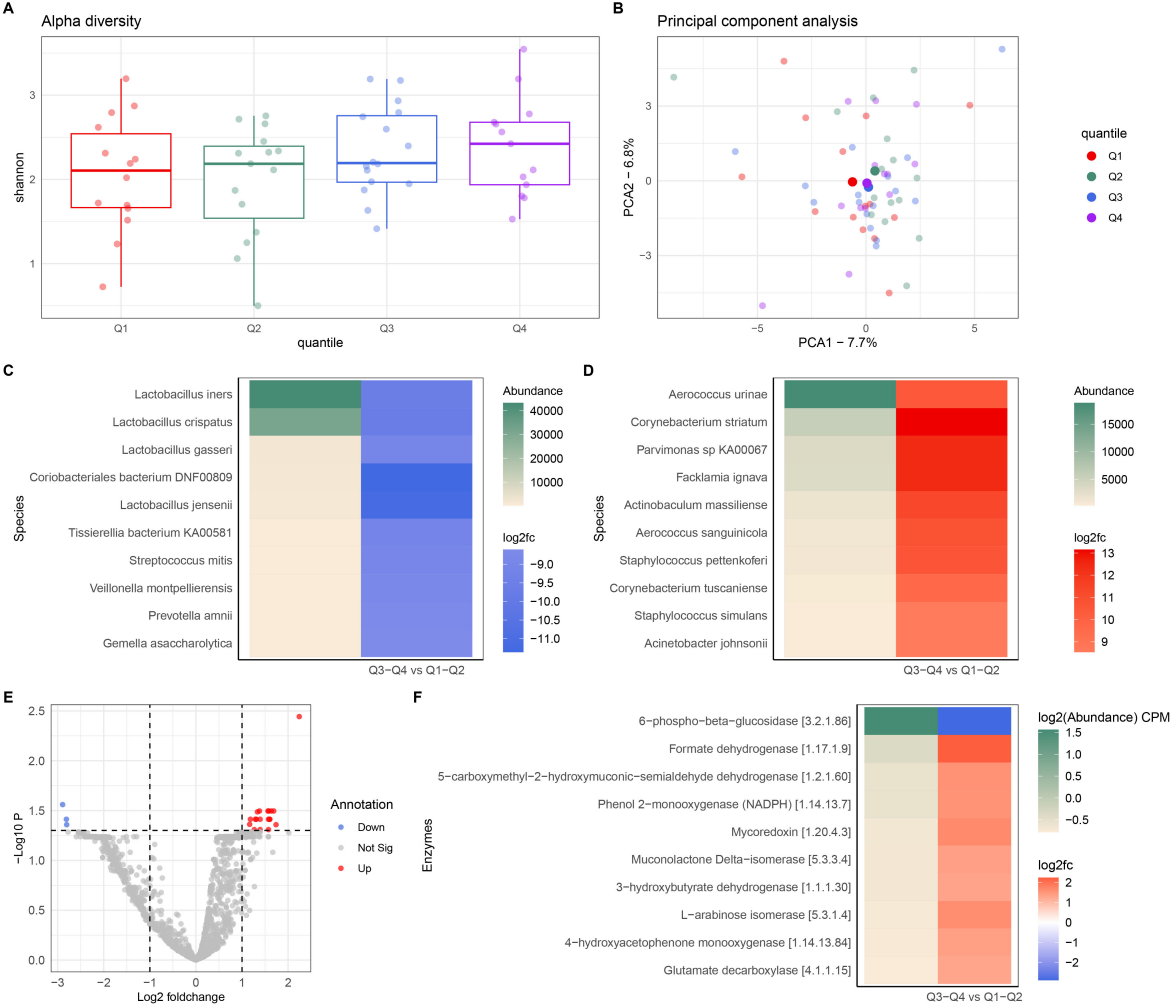
Compositional and functional analysis of healthy vulvar microbiome per age group. **(A)** Shannon diversity index per age group as indicated by statistical quartiles in the cohort. Age distribution per quartiles were Q1: 22–34, Q2: 35–56, Q3: 58–66, and Q4: 67–82 years. **(B)** Principal component analysis (PCA) of healthy vulvar microbiome comparing age quartiles. Individual samples are indicated by a smaller semitransparent dots, whereas mean per quartile is shown by larger non-transparent centroid. **(C)** DAA plot indicating top 10 bacterial species with a reduced abundance in the older group (Q3 + Q4) compared to the younger group (Q1 + Q2). **(D)** DAA plot indicating top 10 bacterial species with an increased abundance in the older group compared to the younger group. **(E)** Vulcano plot presenting differences in the functional capacity of the vulvar microbiome in the older group relative to the younger group. **(F)** DAA showing top 10 differences in functional capacity of the vulvar microbiome in the older group relative to the younger group. Enzyme names and EC identifier codes are shown in the legend. Cohort size *n* = 58.

Vulvar microbiome composition in different age groups was examined by comparing the vulvar microbiomes in women. Based on the age of participants included in the VMLC, quartiles were categorized, which were compared for Shannon diversity (Figure 3A). This showed that the diversity of the vulvar microbiome was comparable among the different age groups. To determine if the composition remains stable during aging, a principal component analysis (PCA) was performed (Figure 3B). The outcomes of the PCA indicated that several species drive variations by age group. Yet, the overall variation per age group was low, with minor variation in the average compositions. To obtain more insights in the variation within the microbiome, a differential abundance analysis (DAA) was performed comparing the vulvar microbiome in the older group (Q3 + Q4) with that of the younger group (Q1 + Q2). This revealed that several bacterial species were less abundant in the older group (Figure 3C), whereas other bacterial species were more abundant in the older group (Figure 3D). Interestingly, multiple species of the *Lactobacillus* genus showed a reduced abundance in the older group as compared to the younger group in the vulvar microbiome. This suggests that despite the low variation between age groups, different abundance of species was detected in our analyses, indicating taxonomic shift in the vulvar microbiome due to age.

Furthermore, we explored the functional capacity of the vulvar microbiomes in different aging groups to elucidate the functional differences at younger and older age. The majority of enzymes were not significantly different, however specific groups of enzymes exhibited variations were different due to aging (Figure 3E). Among the top 10 differences in functional enzymes, nine were found to be more abundant in the vulvar microbiome of older individuals (Figure 3F). These could not be clearly linked to an overarching pathway.

### 3.4 Compositional alteration in the vulvar microbiome associated with high-grade intraepithelial lesions and vulvar lichen sclerosus

**FIGURE 4:**
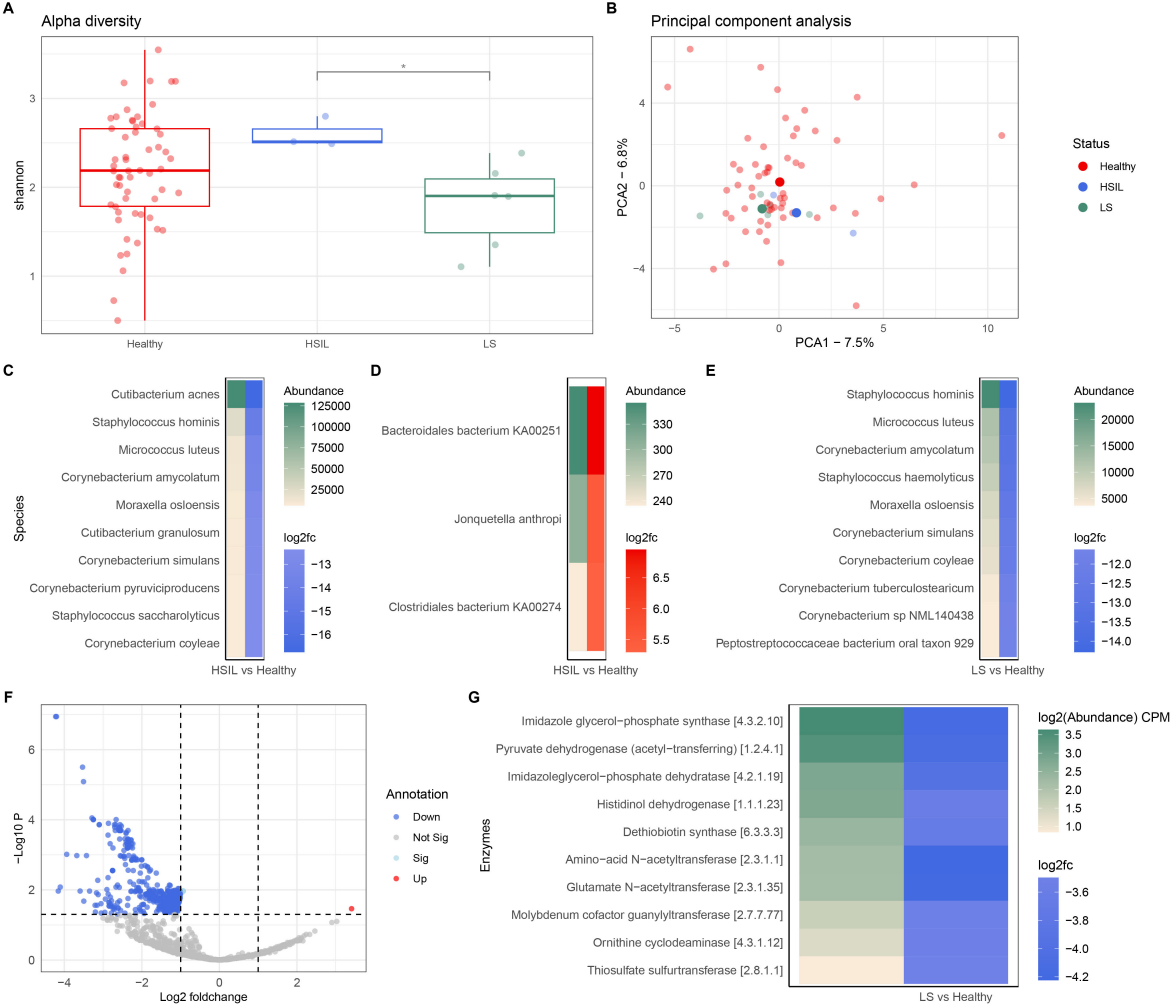
Compositional and functional variations of the vulvar microbiome in health and disease. **(A)** Boxplot of the alpha diversity of the vulvar microbiome in healthy controls (*n* = 58) versus high-grade squamous intraepithelial lesion (HSIL) (*n* = 3), and vulvar lichen sclerosus (LS) (*n* = 6). The asterisk * indicates *p* < 0.05 as a result of Dunn’s test. **(B)** PCA of healthy (in red), HSIL (in blue), and LS (in green) vulvar microbiomes. **(C)** DAA showing top 10 bacterial species that have a reduced abundance in HSIL. DAA is presented by the differential abundance (in green) and log2FC values. **(D)** DAA plot indicating three bacterial species with a higher abundance in HSIL. **(E)** DAA showing top 10 bacterial species that have a reduced abundance in LS. **(F)** Vulcano plot presenting differences in the functional capacity of the vulvar microbiome in LS versus healthy controls. **(G)** DAA showing top 10 differences in functional capacity of the vulvar microbiome in LS. Enzyme names and EC identifier codes are shown in the legend.

To evaluate if the vulvar microbiome was altered in vulvar diseases, a comparison of the vulvar microbiome in healthy conditions to that of individuals with high-grade squamous intraepithelial lesion (HSIL) or individuals with vulvar lichen sclerosus (LS) was performed. In HSIL, the pathogenesis in the majority of cases is linked to human papillomavirus (HPV)-associated lesions, although there are HPV-independent lesions which have a distinct risk for disease progression (Thuijs et al., 2024). The etiology of non-infectious vulvar LS remains unknown, although current hypotheses are based on autoimmunity, genetics, local infection, and/or hormonal etiology, with a multifactorial origin composed of these elements as main hypothesis (Singh and Ghatage, 2020). The vulvar microbiome in women with HSIL or LS was compared for alpha diversity to the healthy group, which showed no difference (Figure 4A). Yet, in HSIL the diversity of the microbiome was high and reached significance compared to LS, although due to the small number of samples within this group this should be investigated in larger cohorts. Comparative analyses were also performed with PCA, which revealed small differences between healthy control, HSIL, and LS vulvar microbiomes, namely variation on PC1 of 7.5% and PC2 of 6.8% (Figure 4B). Taxonomic variations were analyzed by DAA. This revealed that multiple bacterial species were present in a lower relative abundance (Figure 4C) or higher relative abundance (Figure 4D) in HSIL as compared to healthy control. In the DAA comparing the vulvar microbiome of LS to that of healthy controls, several species were present in a lower relative abundance, whereas no bacterial species were present in a higher relative abundance (Figure 4E). Several species showed reduced abundance in both HSIL and LS (*e.g.*, *Staphylococcus hominis*, *Micrococcus luteus*, *Moraxella osloensis*, and *Corynebacterium simulans*). In addition, the functional capacity of the vulvar microbiome of individuals with LS was compared to that of healthy controls. Most functional pathways were downregulated in LS (Figure 4F), of which the top differences in enzymes are presented in Figure 4G. Three enzymes in the top 10 are linked to the L-histidine pathway (EC [4.3.2.19], [4.2.1.19], and [1.1.1.23]). Due to the low number of subjects with HSIL (*N* = 3), evaluation of functional capacity was not performed.

## 4 Discussion

In this study, we present the characterization of the vulvar microbiome in a cohort of 67 individuals on species level taxonomy and functional profiling with shotgun metagenomic sequencing. We showed that the vulvar microbiome can be categorized as skin-, vagina-, or multispecies mixture- dominant bacterial signature, based on species predominantly present in the microbiome of the respective tissues. The effect of aging appeared minor, though there were a few species (e.g., *Lactobacillus* spp.) and enzymes identified that were different in abundance upon aging. The microbiome of women with a vulvar disease, either HSIL or LS, also showed small variations, as multiple species and functional enzymes (e.g., L-histidine pathway) were different in abundance as compared to healthy controls.

Our findings are in line with the observations of previous analyses (Pagan et al., 2021), where the results in ten different studies (Brown et al., 2007; Costello et al., 2009; Shiraishi et al., 2011; Miyamoto et al., 2013; Jayaram et al., 2014; Hickey et al., 2015; Vongsa et al., 2019; Bruning et al., 2020; Murina et al., 2020; Chattopadhyay et al., 2021) were reviewed. The authors (Pagan et al., 2021) concluded that the vulvar microbiome is characterized by the presence of several taxa of the *Lactobacillus*, *Corynebacterium*, *Staphylococcus* and *Prevotella* genera, which can form a unique niche or can be emerged from vaginal, cutaneous, and/or fecal origin. We did not observe a fecal microbiome signature in our data analysis. It is not clear whether earlier observations were affected by contamination or due to variability in sampling location or methodology. However, in all previously published studies, taxonomy was determined by 16S sequencing. In contrast, we provide novel insights by taxonomic profiling at the species level by using shotgun metagenomic sequencing. This allowed us to classify the vulvar microbiome as skin- dominant microbiome in 24% of participants (14 out of 58), as vagina- dominant microbiome in 22% of participants (13 out of 58), or as multispecies mixture- dominant microbiome in 53% (31 out of 58) of participants, which all showed differences in their functional capacities. In the vulvar microbiome with a vagina- dominant signature, a different functional capacity regarding the aspartate pathway was detected, which is a metabolite that is downregulated upon dysbiosis in the vaginal microbiome (Gill et al., 2023). Nevertheless, its role in the vulvar microbiome is not yet uncovered, indicating that our observations warrant further research, as the microbiome-host interactions, microbial metabolome, and microbe-microbe interactions for the vulva and the vulvar microbiome are still understudied.

The intrinsic factor of aging has been explored in our cohort, as aging is a key factor impacting the composition of the microbiome. The onset of menopause and alterations in hormones such as a decrease in estrogen levels have been reported to affect microbiome composition in the vagina (Park et al., 2023) and in the gut (Peters et al., 2022). This is characterized by a reduced presence of *Lactobacilli* and an increase in local pH of the vagina, whereas the diversity of the gut microbiome was reduced. Our results do not show large shifts in the diversity of the vulvar microbiome, although it is highly interesting that several *Lactobacillus* species were more abundant in the younger individuals of the cohort. This is in line with alterations in diversity reported by aging and menopause in the vaginal microbiome (Greenbaum et al., 2019; Walsh et al., 2019). More in-depth exploration of aging and lifestyle factors should be facilitated by larger, population-scale intimate zone microbiome studies.

In vulvar diseases, the microbiome has been sparsely studied. An exploratory study was performed by Pyle et al. (2024), which identified associations between the vulvar microbiome and LS in postmenopausal females. Although our cohort does not report menopausal states, the age distribution is younger on average compared to that of Pyle et al. (2024). Despite that difference, we also report differences in the composition of the vulvar microbiome in LS as compared to healthy individuals, which requires further investigation. A substantial contribution of the host epithelium on the orchestration of the vulvar microbiome is expected, as hormonal levels were associated with LS (Pyle et al., 2024). We describe that the functional capacity regarding the L-histidine pathway was the most altered in LS compared to healthy individuals. It is of interest to obtain more insights on the role of the L-histidine pathway in the vulvar ecosystem, especially since therapeutic implications of the L-histidine pathway are explored in other conditions (Feng et al., 2013; Quesada-Vázquez et al., 2023). In individuals with cervical HSIL, the vaginal microbiome has been determined previously by Mitra et al. (2015). In that study, a higher bacterial diversity was detected in HSIL as compared to controls. Although in our cohort we only have 3 subjects included, and we did sample from another epithelial surface, our observations in HSIL also demonstrated a relatively high microbial diversity.

Limitations of this study included a smaller sample size within each subgroup of women with a vulvar disease and the sampling was conducted at a single time point. Moreover, potential confounders that can affect the composition and functional capacity of the vulvar microbiome should be evaluated in meta-analyses and in future cohort studies, to cover more in-depth biological variations (e.g., hormonal status, hormonal dynamics, and lifestyle) and technical variations (e.g., sampling techniques, sequencing methods, and bioinformatical analyses). These limitations, especially on the cohort size, utilization of a single DAA tool (DESeq2), and potential contribution of background microbe detection, accentuate the exploratory nature of this project, which should be aimed to overcome in future studies. To better understand dynamics within the vulvar microbiome or the role of the vulvar microbiome during onset and progression of diseases, larger cohorts and more sampling time points are needed. Furthermore, to assign a skin- or vagina- dominant signature, we selected a limited number of species associated with these tissues, thereby partially omitting species with a natural low abundance level. Another limitation is that an inter-kingdom analysis was not performed, also covering relative abundance of the vulvar virome, mycobiome, parasitome, and archaeome. An inter-kingdom analysis would enhance our understanding of the vulvar ecosystem in healthy and diseased states.

In conclusion, by performing shotgun metagenomic sequencing, we were able to unravel novel insights in the composition and functional capacity of the vulvar microbiome, enhancing our understanding of its role in women’s health and disease. We identified variations in microbial taxa and functional capacities associated with age and disease, classifying the vulvar microbiome at the individual level into skin-, vagina-, or multispecies mixture- dominant bacterial signatures, each with distinct functional capabilities. Further research is urgently needed to elucidate how these variations impact women’s overall health.

## Data Availability

The data and metadata as presented in this study are deposited in the European Nucleotide Archive (https://www.ebi.ac.uk/ena/browser/home), accession number PRJEB85994.

## Supplementary material

Supplementary Table 1 Overview of sample sequencing and metadata.

Supplementary Table 2 Overview of microbiome composition in the vulvar multispecies mix other subgroup.

Supplementary Figure 1 Taxonomic analysis of healthy vulvar microbiome per group. **(A)** Principal component analysis (PCA) of CHDR and TNO vulvar microbiome samples. Individual samples are indicated by a smaller semi transparant dots, whereas mean per condition is shown by larger non-transparant centroid. Statistical testing was performed with PERMANOVA, showing non-significant outcomes in p.values and p.adjusted values presented in the table. **(B)** Alpha diversity index of the vulvar microbiome in healthy participants. Comparative analysis of Shannon diversity index in the CHDR (*N* = 9) and the TNO (*N* = 49) cohorts were statistically tested by Dunn’s test and found non-significant different (p.adjusted = 0.0574).

Supplementary Figure 2 Taxonomic and compositional analysis of healthy vulvar microbiome per subgroup. **(A)** Boxplot of the alpha diversity of the vulvar microbiome grouped by signatures. Significance was tested by Dunn’s test and results presented as **, and *** indicating p.adjusted < 0.01, and 0.001, respectively. **(B)** Principal component analysis (PCA) of healthy vulvar microbiome comparing per group. Individual samples are indicated by a dots and colored by group. Statistics were performed by PERMANOVA (Skin vs. Other: p.adjusted = 0.012, Vagina vs. Other: p.adjusted = 0.831, and Vagina vs. Skin: p.adjusted = 0.012). **(C)** PCA plot representing the top 10 loadings that overlap within the other subgroup. **(D)** PCA plot representing the top 10 loadings that overlap within the skin subgroup. **(E)** PCA plot representing the top 10 loadings that overlap within the vagina subgroup.
