# Supplementary figures and images for: Taxonomic and functional profiling of the vulvar microbiome indicates variations related to ecological signatures, aging, and health status
Source: Front Microbiol. 2025 Sep 19;16:1633147. doi: 10.3389/fmicb.2025.1633147 (PMC12491970; doi:10.3389/fmicb.2025.1633147)

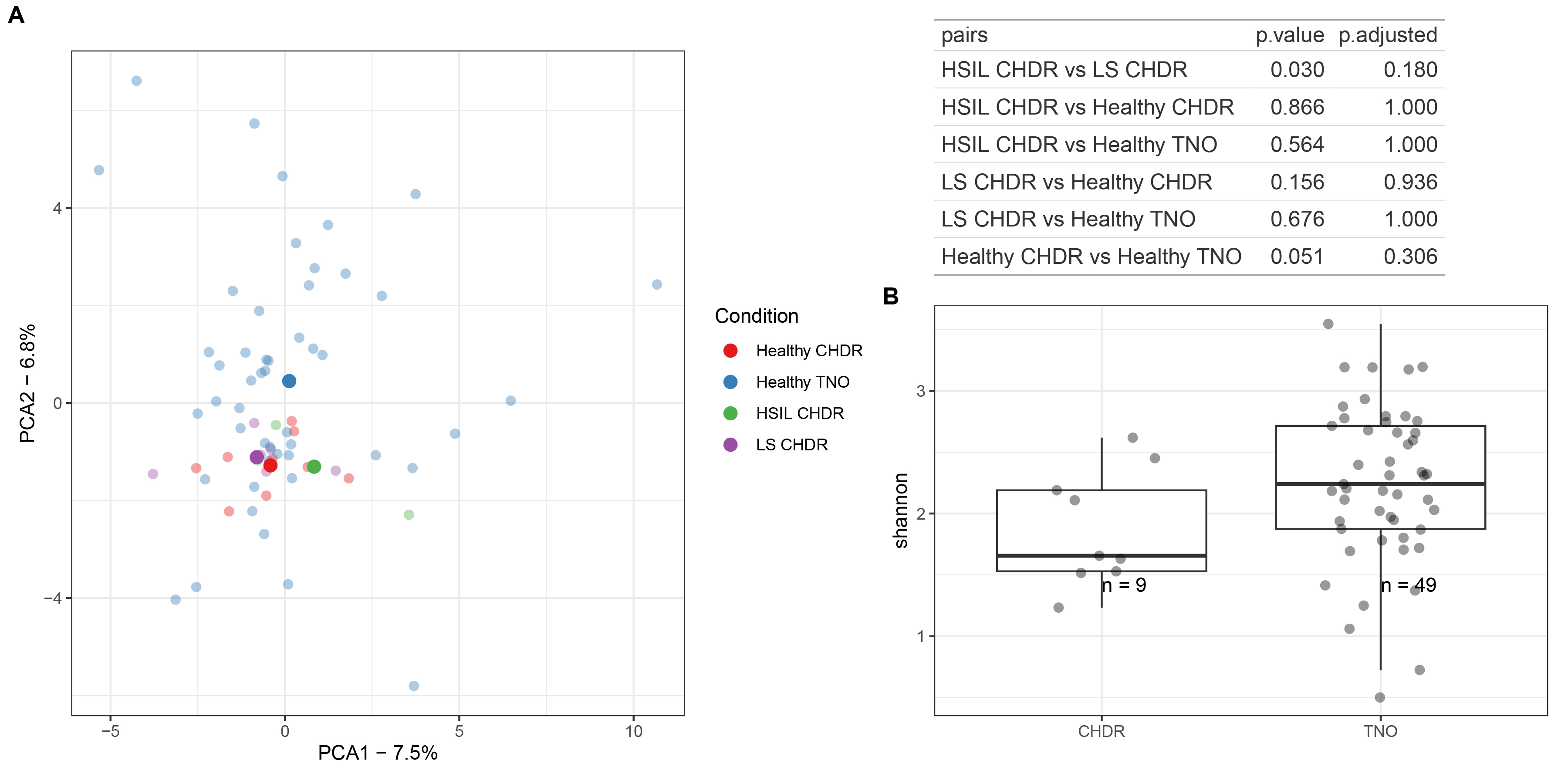

Supplement: Supplementary Figure 1 — Taxonomic analysis of healthy vulvar microbiome per group. (A) Principal component analysis (PCA) of CHDR and TNO vulvar microbiome samples. Individual samples are indicated by a smaller semi transparant dots, whereas mean per condition is shown by larger non-transparant centroid. Statistical testing was performed with PERMANOVA, showing non-significant outcomes in p.values and p.adjusted values presented in the table. (B) Alpha diversity index of the vulvar microbiome in healthy participants. Comparative analysis of Shannon diversity index in the CHDR (N = 9) and the TNO (N = 49) cohorts were statistically tested by Dunn’s test and found non-significant different (p.adjusted = 0.0574). [file Image_1.jpeg]

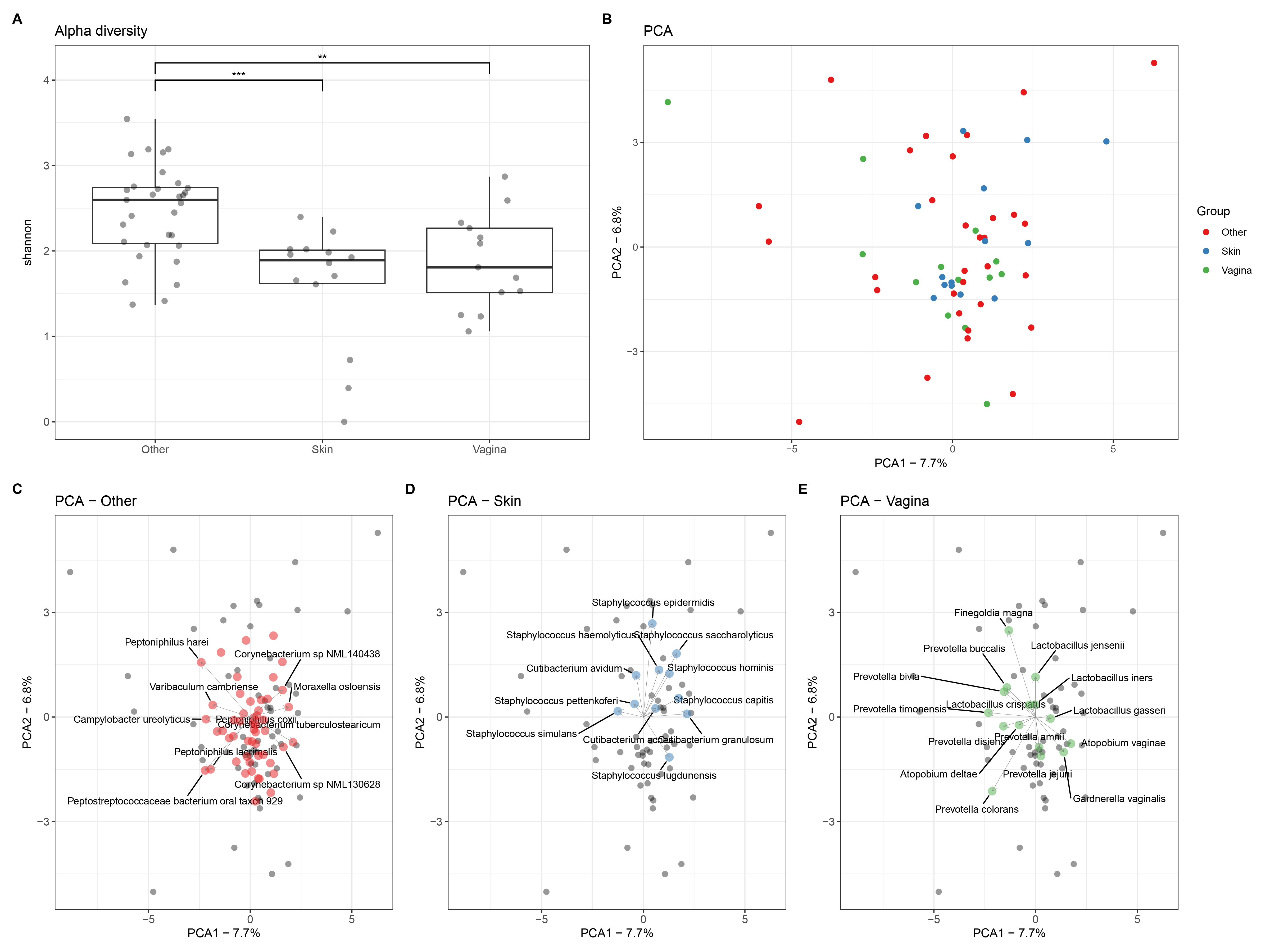

Supplement: Supplementary Figure 2 — Taxonomic and compositional analysis of healthy vulvar microbiome per subgroup. (A) Boxplot of the alpha diversity of the vulvar microbiome grouped by signatures. Significance was tested by Dunn’s test and results presented as **, and *** indicating p.adjusted < 0.01, and 0.001, respectively. (B) Principal component analysis (PCA) of healthy vulvar microbiome comparing per group. Individual samples are indicated by a dots and colored by group. Statistics were performed by PERMANOVA (Skin vs. Other: p.adjusted = 0.012, Vagina vs. Other: p.adjusted = 0.831, and Vagina vs. Skin: p.adjusted = 0.012). (C) PCA plot representing the top 10 loadings that overlap within the other subgroup. (D) PCA plot representing the top 10 loadings that overlap within the skin subgroup. (E) PCA plot representing the top 10 loadings that overlap within the vagina subgroup. [file Image_2.jpeg]
